# Perspectives of tissues in silico

**DOI:** 10.17179/excli2015-219

**Published:** 2015-03-11

**Authors:** Seddik Hammad, Mosaab A. Omar, Mohammed F. Abdallah, Hassan Ahmed

**Affiliations:** 1 Department of Forensic Medicine and Veterinary Toxicology, Faculty of Veterinary Medicine, South Valley University, 83523 Qena-Egypt; 2 Department of Medical Laboratories, Collage of Applied Medical Sciences, Majmaah University-Kingdom of Saudi Arabia; 3 Department of Pharmaceutical Toxicology, Faculty of Pharmacy, Hacettepe University, S?hyyie/Ankara-Türkiye; 4 Division of Cerebral Circuitry, National Institute for Physiological Sciences, Okazaki-Japan

## ‬‬‬‬

Over the past decade much effort has been invested into the development of *in vitro* systems as alternatives to animal experiments (Hammad et al., 2013[15], 2014[12]; Hammad, 2013[11]; Godoy et al., 2010[9], 2013[8]; Hewitt et al., 2007[17]; Stewart and Marchan, 2012[25]; Gebel et al., 2014[6]; Grinberg et al., 2014[10]). However, *in vitro* systems still have the limitation that they often do not sufficiently represent the in vivo situation. Moreover, quantitative *in vitro* to *in vivo* extrapolation is difficult (Ghallab, 2013[7]; Reif, 2014[21]; Stewart, 2010[24]). 

In recent years a concept is emerging that may overcome many of the current limitations of *in vitro* testing, namely *in silico* tissues (Hoehme et al., 2010[18]; Schliess et al., 2014[22]). Typically, virtual tissues are based on reconstructions of real tissues, where the exact positions of each individual cell and further relevant structures, e.g. blood vessels, are known in a three-dimensional space (Hoehme et al., 2010[18]; Höhme et al., 2007[19]). In the first step spatio-temporal models are generated from reconstructions (Hammad et al., 2014[14]). For this purpose the individual cell serves as the smallest unit. Model parameters, such as the probability to divide or to die, and even more complex properties, such as migration rules can be programmed into each cell. This results in a model that can simulate, for example, the spatio-temporal process of tissue damage and regeneration. Key principles how cells in the liver coordinately respond to large destructions to restore functional tissue have been identified by such models (Drasdo et al., 2014[3]; Hoehme et al., 2010[18]). In next steps, further processes can be integrated into spatio-temporal models, e.g. blood flow or metabolic processes. As an example, Schliess et al. (2014[22]) have integrated metabolic pathway models of ammonia detoxification into spatio-temporal models. This allows simulating ammonia concentrations in the blood circulation and how they are influenced by specific damage patterns of the liver.

In toxicology, modelling especially structure activity and physiologically-based-pharmacokinetic (PBPK) models have a long standing tradition (Schug et al., 2013[23]; Karamanakos et al., 2009[20]; Carlsson et al., 2004[1]; Thiel et al., 2015[26]; Hammad and Ahmed, 2014[13]; Dobrev et al., 2001[2]; El-Masri et al., 1996[5]). However, the advent of spatio-temporal models with the possibility to integrate other model types opens new possibilities. Integrated mathematical models formalize the relationship between individual components to test their interactions in a virtual setting (Drasdo et al., 2014[3][4]; Widera, 2014[27]). It can be expected that virtual tissue approaches will have a strong impact to understand complex pathophysiologies, especially when processes and interactions have to be elucidated that cannot be directly measured by established methods.
